# Sequential and Hybrid PET/MRI Acquisition in Follow-Up Examination of Glioblastoma Show Similar Diagnostic Performance

**DOI:** 10.3390/cancers15010083

**Published:** 2022-12-23

**Authors:** Julian Ziegenfeuter, Claire Delbridge, Denise Bernhardt, Jens Gempt, Friederike Schmidt-Graf, Michael Griessmair, Marie Thomas, Hanno S. Meyer, Claus Zimmer, Bernhard Meyer, Stephanie E. Combs, Igor Yakushev, Benedikt Wiestler, Marie-Christin Metz

**Affiliations:** 1 Department of Neuroradiology, Klinikum Rechts der Isar, TU Munich, 81675 München, Germany; 2 Department of Pathology, TU Munich, 81675 München, Germany; 3 Department of Radiation Oncology, Klinikum Rechts der Isar, TU Munich, 81675 München, Germany; 4 Department of Neurosurgery, Klinikum Rechts der Isar, TU Munich, 81675 München, Germany; 5 Department of Neurosurgery, University Medical Center Hamburg-Eppendorf, 20251 Hamburg, Germany; 6 Department of Neurology, Klinikum Rechts der Isar, TU Munich, 81675 München, Germany; 7 Department of Nuclear Medicine, Klinikum Rechts der Isar, TU Munich, 81675 München, Germany; 8 TranslaTUM, TU Munich, 81675 München, Germany

**Keywords:** glioblastoma, PET, DSC perfusion, treatment-related changes

## Abstract

**Simple Summary:**

Reliable differentiation between true tumor progression and treatment-related changes is a challenging situation in the management of glioma patients. Both amino-acid PET and perfusion MRI, as well as their combination, play a central role in this decision. In clinical practice, PET and MRI are usually acquired at two separate time points, so the question arises if and how this affects their diagnostic performance. In our study, we investigated a unique cohort of 38 glioblastoma patients (*IDH* wild-type), who received both a PET–MRI (with simultaneous acquisition of FET-PET and DSC perfusion) as well as an MRI exam with DSC perfusion within a month of each other. For all global and local image metrics, and importantly also for the diagnostic performance, we found no significant difference between the simultaneous and sequential acquisition of PET and MRI. These results are reassuring for routine clinical management and support further investigation into advanced, multi-parametric models for improving personalized decision-making in neuro-oncology when PET and MRI are not acquired simultaneously.

**Abstract:**

Both positron emission tomography (PET) and magnetic resonance imaging (MRI), including dynamic susceptibility contrast perfusion (DSC-PWI), are crucial for treatment monitoring of patients with high-grade gliomas. In clinical practice, they are usually conducted at separate time points. Whether this affects their diagnostic performance is presently unclear. To this end, we retrospectively reviewed 38 patients with pathologically confirmed glioblastoma (*IDH* wild-type) and suspected tumor recurrence after radiotherapy. Only patients who received both a PET–MRI (where DSC perfusion was acquired simultaneously with a FET-PET) and a separate MRI exam (including DSC perfusion) were included. Tumors were automatically segmented into contrast-enhancing tumor (CET), necrosis, and edema. To compare the simultaneous as well as the sequential DSC perfusion to the FET-PET, we calculated Dice overlap, global mutual information as well as voxel-wise Spearman correlation of hotspot areas. For the joint assessment of PET and MRI, we computed logistic regression models for the differentiation between true progression (PD) and treatment-related changes (TRC) using simultaneously or sequentially acquired images as input data. We observed no significant differences between Dice overlap (*p* = 0.17; paired *t*-test), mutual information (*p* = 0.18; paired *t*-test) and Spearman correlation (*p* = 0.90; paired *t*-test) when comparing simultaneous PET–MRI and sequential PET/MRI acquisition. This also held true for the subgroup of patients with >14 days between exams. Importantly, for the diagnostic performance, ROC analysis showed similar AUCs for differentiation of PD and TRC (AUC simultaneous PET: 0.77; AUC sequential PET: 0.78; *p* = 0.83, DeLong’s test). We found no relevant differences between simultaneous and sequential acquisition of FET-PET and DSC perfusion, also regarding their diagnostic performance. Given the increasing attention to multi-parametric assessment of glioma treatment response, our results reassuringly suggest that sequential acquisition is clinically and scientifically acceptable.

## 1. Introduction

Glioblastoma is the most common primary brain tumor in adults [1]. Despite multimodal therapy, including maximal safe resection and consecutive radiochemotherapy, tumor recurrence is almost inevitable and the prognosis remains extremely poor [2]. A particularly challenging situation in the management of glioblastoma patients is the differentiation between true tumor progression (progressive disease, PD) and treatment-related changes (TRC). Despite various pathophysiological differences, conventional MRI signal behavior is quite similar [3]. Since both can lead to mass effect, perilesional edema, and contrast enhancement, TRC may mimic PD, a major issue in therapy monitoring and clinical decision-making [4].

Previously performed therapies can result in multiple non-tumorous processes, e.g., ischemia, postsurgical changes, treatment-related inflammation, subacute radiation effects, and radiation necrosis [5]. These alterations can disrupt the blood–brain barrier and consequently appear as increased contrast enhancement on T1-weighted gadolinium-enhanced MRI. Since these lesions also may exert mass effect, they often mimic tumor growth. In contrast, PD-related increased contrast enhancement is usually the result of angiogenesis and neovascularization—a hallmark of malignant gliomas [6]. The question of whether there is PD or TRC is clinically highly important since these two entities have radically different treatment approaches and prognosis [4]. Despite its wide usage, a conventional MRI protocol (clinical standard), including fluid-attenuated inversion recovery (FLAIR), T2-weighted, and T1-weighted sequences before and after contrast injection, does not allow a reliable distinction between PD and TRC [7]. Various additional functional imaging modalities such as perfusion-weighted imaging (PWI) and amino acid positron emission tomography (PET) go beyond the diagnostic value of standard anatomic imaging and can provide insight into tumor physiology and key oncogenic processes. 

Dynamic susceptibility contrast perfusion (DSC-PWI), a dynamic T2*-weighted sequence, measures the brain signal intensity before, during, and after contrast injection to calculate regional brain perfusion parameters such as relative cerebral blood volume (rCBV) [8]. PET is a nuclear medicine method that, in the case of glioblastoma, typically uses O-(2-18F fluoroethyl)-L-tyrosine (18F-FET) to detect amino acid uptake in malignant cells in order to visualize tumor metabolism [9]. 

The Congress of Neurological Surgeons recently published newly updated guidelines on the role of imaging in the management of progressive glioblastoma in adults. Both PET with amino acid agents as well as DSC-PWI are recommended with level III evidence [10]. Accordingly, several meta-analyses demonstrated the value of both DSC-PWI as well as FET-PET for differentiating between PD and TRC in high-grade glioma [11,12]. Since these modalities provide complementary information about neoangiogenesis and proliferation, combining both is superior to the single acquisition of either modality alone [13,14], in particular, when modern machine learning techniques are used to integrate information [15].

In clinical practice, the availability of hybrid PET–MRI scanners is often limited, and therefore FET-PET and DSC-PWI are usually acquired at two separate time points. However, it is currently unclear whether the sequential acquisition of PET and MRI influences their diagnostic performance, for example, due to interim tumor growth or technical aspects such as imprecise co-registration of both modalities. In this study, we used a local cohort of 38 *Isocitrate Dehydrogenase* (*IDH*) wild-type glioblastoma patients with suspected tumor recurrence who received both a PET–MRI (including a simultaneous DSC-PWI) as well as a DSC-PWI at a different time point for evaluation of PD vs. TRC. Our goal was to investigate possible differences in diagnostic significance between simultaneous and sequential PET–MRI and DSC-PWI acquisition to guide clinicians in their decisions for optimal patient care.

## 2. Materials and Methods

### 2.1. Patient Selection

We retrospectively reviewed patients with suspected tumor recurrence and pathologically confirmed glioblastoma (*IDH* wild-type, WHO Grade 4 according to the 2021 World Health Organization (WHO) classification of Central Nervous System tumors [16]) who received maximal safe resection followed by radiochemotherapy via Stupp protocol [17]. We included 38 patients with an available PET–MRI (with simultaneous acquisition of FET-PET and DSC perfusion) as well as an MRI exam with DSC perfusion, either before or after the PET–MRI, for the differentiation between PD and TRC. All exams were taken before any change of therapy was initiated. All examinations needed to contain FLAIR, T2w, 3D-T1w, 3D-T1w post-contrast sequences for automated tumor segmentation. In the case of missing sequences, we employed a generative adversarial network (GAN) to synthesize the missing sequences as described earlier [18]. In this cohort, we needed to synthesize two missing T2w images. The time distance between baseline MRI and baseline 18F-FET-PET scan was determined to be a maximum of 6 weeks. For final diagnostic confirmation of PD or TRC, we either used a follow-up MRI with 12 weeks’ time interval between baseline and follow-up or histopathological analysis after repeat biopsy or resection when available. All images were rated according to the Response Assessment in Neuro-Oncology (RANO) criteria [19].

### 2.2. Imaging Data

The majority of MR imaging was acquired on a Philips (Best, The Netherlands) 3 Tesla whole-body scanner (n = 36) (Achieva or Ingenia) or on a Siemens Verio (Siemens Healthcare GmbH, Erlangen, Germany) 3 Tesla whole-body scanner (n = 2). The Philips protocol includes an isotropic FLAIR (voxel size 1 mm^3^, Echo Time (TE) = 269 ms, Repetition Time (TR) = 4800 ms, Inversion Time (TI) = 1650 ms), isotropic T1w Turbo Field Echo (TFE) (voxel size 1 mm^3^, TE = 4 ms, TR = 9 ms) before and after contrast, axial T2w (voxel size 0.36 × 0.36 × 4 mm, TE = 87 ms, TR = 3396 ms), DSC perfusion (voxel size 1.75 × 1.75 × 4 mm, TE = 40 ms, TR = 1547 ms, Flip Angle = 75°, 80 dynamics).

The 18F-FET PET scans were obtained with a PET/MR scanner (Biograph mMR, Siemens Healthcare GmbH, Erlangen, Germany), according to a standard clinical protocol. We asked patients to fast for a minimum of 4 h before undergoing scanning. Emission scans were acquired at 30 to 40 min after intravenous injection of a target dose of 185 ± 10% MBq 18F-FET. Attenuation correction was performed according to the vendor’s protocol.

### 2.3. Image Analysis

All images from a single time point as well as the PET scan were rigidly co-registered into the SRI24 atlas space using NiftyReg [20]. Using the freely available BraTS.Toolkit [21], the tumors were automatically segmented into necrosis, contrast-enhancing tumor (CET), and edema. All registrations and segmentations were manually inspected and corrected where necessary.

Maps of normal-appearing white matter (NAWM) were generated using ANTs Atropos [22]. For the estimation of leakage-corrected and normalized cerebral blood volume (CBV) maps from the raw DSC data, we employed a previously published method by Arzanforoosh [23]. Tumor-background-ratio normalization of PET images also used the NAWM maps for background intensity calculation.

From these coregistered and segmented PET and CBV maps, we extracted Dice overlap of hotspot areas in PET (tumor-background-ratio (TBR) > 2.0) and CBV (rCBV > 1.5) in contrast-enhancing tumor and peritumoral edema, voxel-wise Spearman correlation as well as normalized mutual information (using a default n_bins = 32) [24].

### 2.4. Statistical Analysis

To compare the diagnostic accuracy of combined analysis of PET and CBV maps for differentiation of PD and TRC, we calculated logistic regression models using both the mean and maximum signal of FET and CBV maps in contrast-enhancing the tumor. The resulting areas-under-the-curve (AUC) were compared using DeLong’s test. Image metrics between simultaneous and sequential PET/MRI data were compared using a paired *t*-test, given the paired nature of the images. Image processing and statistical analyses were conducted in Python (v 3.8.10) and GraphPad PRISM (v 9.4.1, GraphPad, San Diego, CA, USA).

## 3. Results

### 3.1. Patient Cohort

**Figure 1:**
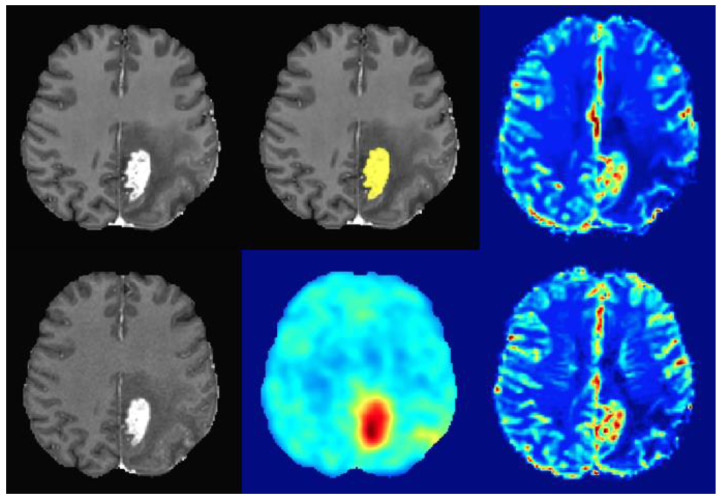
Exemplary images of a 57-year-old female patient with left parietal glioblastoma (upper left: MRI contrast-enhanced T1, upper middle: MRI contrast-enhanced T1 with automated segmentation overlay (yellow: CET), upper right: MRI CBV, lower left: PET/MRI contrast-enhanced T1, lower middle: FET-PET, lower right: PET/MRI CBV).

Overall, 38 patients fulfilled our inclusion criteria. The median age was 59 years with 55% male (n = 21) and 45% female (n = 17) patients. The median interval between MRI and sequential PET/MRI was 13.5 days (range: 1–41 days). 28 patients with PD and 10 patients with TRC were diagnosed using histopathology (n = 27) or follow-up MRI (n = 11) as the reference standard. Figure 1 shows representative images of a patient with glioblastoma.

### 3.2. Spatial Overlap, Mutual Information, and Spearman Correlation of Imaging Hotspots

**Figure 2:**
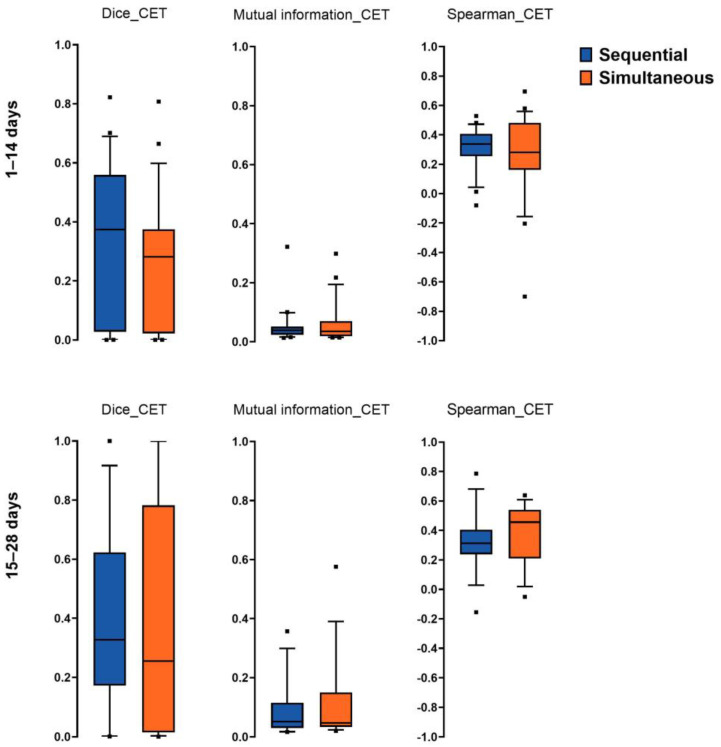
Dice score (Dice), mutual information and Spearman correlation (Spearman) of contrast-enhancing tumor areas (CET). No significant difference was observable in the 90th percentile between sequential (blue) and simultaneous PET/MRI (orange) image acquisition for group 1 (1–14 days; n = 22) as well as for group 2 (15–28 days; n = 14). Dots denote outliers.

We found no significant differences between Dice overlap in contrast-enhancing tumor areas (*p* = 0.17; paired *t*-test) and areas segmented as perilesional edema (*p* = 0.10; paired *t*-test) when comparing simultaneous PET–MRI and sequential PET/MRI acquisition. This held true also for global mutual information (*p* = 0.18; paired *t*-test) and Spearman correlation (*p* = 0.90; paired *t*-test) for CET as well as for edema (mutual information: *p* = 0.95; paired *t*-test and Spearman correlation: *p* = 0.39; paired *t*-test) (Figure 2).

We further investigated the influence of the time difference of sequential PET/MRI acquisition. Therefore, we divided all patients scanned within 28 days into two groups using 14 days as a cut-off for the time difference. We again found no significant differences either in patient group 1 (1–14 days; n = 22 patients) or patient group 2 (15–28 days; n = 14 patients) when comparing the Dice overlap, mutual information and Spearman correlation for CET (Figure 2).

### 3.3. Diagnostic Power of Sequential PET/MRI Acquisition

**Figure 3:**
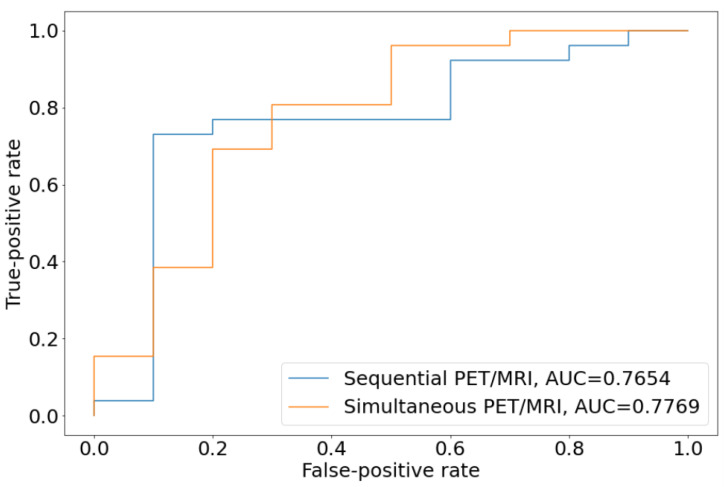
Receiver operating characteristics (ROC) curve analysis showed similar AUCs for the differentiation of PD and TRC. The orange line represents the ROC curve for simultaneous PET/MRI, the blue line represents the ROC curve for sequential PET/MRI (*p* = 0.83, DeLong’s test.).

There was no significant difference in the diagnostic accuracy of sequential vs. simultaneous PET/MRI acquisition in logistic regression analysis (*p* = 0.83; DeLong’s test). Sequential PET/MRI showed a decent prognostic power for the differentiation of PD and TRC with an AUC of 0.77 (95% CI: 0.582–0.971), which was comparable to simultaneously acquired PET–MRI with an AUC of 0.78 (95% CI: 0.559–0.962) (Figure 3).

## 4. Discussion

Especially for challenging clinical situations such as therapy monitoring of glioblastoma patients, there is an urgent need for reliable non-invasive methods to assess tumor biology in order to draw correct conclusions and avoid misinterpretations [4]. The unbiased integration of multimodal imaging information provides significant details crucial for personalized treatment decisions. In a clinical routine, FET-PET and MRI acquisitions are usually performed sequentially [25]. In this work, we aimed to understand the potential differences in the simultaneous and sequential acquisition of FET-PET and DSC-MRI in patients with glioblastoma and suspected tumor recurrence after standard therapy. 

Reassuringly for the clinical routine, we found no significant difference between both, in particular when investigating global and local image metrics such as Dice overlap, mutual information, and Spearman correlation in CET. This held true also when separately analyzing the time difference between group 1 (1–14 days delay between PET and MRI) and group 2 (14–28 days). Importantly, our logistic regression models revealed similar AUCs for the differentiation between true tumor progression and treatment-related changes, using both acquisition strategies as input data and highlighting that both simultaneous, as well as sequential acquisition, convey the same clinical information.

The synergistic value of multiple imaging techniques for response assessment as well as diagnostic performance for patients with glioma has been evaluated by several groups. A recent study retrospectively investigated the diagnostic performance of sequential DSC-MRI perfusion and dynamic 18F-FET PET in terms of PD and TRC in gliomas. Here, the authors showed that a complemental (and thus sequential) use of PWI and 18F-FET PET for the differentiation of PD and TRC in gliomas gives the highest diagnostic accuracy [13].

Brendle et al. have investigated the diagnostic performance of 18F-FET PET/MRI hybrid scanner and its effect on clinical management. For newly diagnosed brain tumor patients they concluded a superior diagnostic performance of multiparametric 18F-FET PET/MRI to that of every single modality alone. Considering adding static 18F-FET PET to an already existing MRI examination seems to be of equal value [25].

As previously noted, the added value of combined 18F-FET PET and MRI has been well demonstrated. However, to the best of our knowledge, a possible difference in the diagnostic effect between simultaneous and sequential acquisition has never been investigated, in particular for such a unique patient cohort. 

Proofing that there is no diagnostic disadvantage in a sequential PET/MRI acquisition ameliorates the urgency on clinicians to force a rapid PET/MRI examination for their patients, especially in centers where hybrid scanners are not easily available. In addition to that, it might be even beneficial to perform a sequential PET/MRI exam because of the accumulated scan time when performing both modalities at once.

Modern, advanced MRI protocols usually include techniques such as diffusion imaging (diffusion tensor or diffusion-weighted imaging), perfusion-weighted imaging, MR spectroscopy, and amide proton transfer-weighted imaging. Adding all scan times of those advanced sequences to the duration of the PET scan, might result in unreasonable follow-up examination time, especially when dealing with a fragile patient cohort. Long scan times further come with an increased likelihood of movement artifacts and reduced compliance of the patients.

In line with a study from Schön et al., who, in contrast to our study, investigated newly diagnosed glioma, we observed a relevant spatial overlap (Dice score) between CBV and FET in CET, indicating their synergistic biological value [26].

### Limitations

Although our results are promising, the present study contains some limitations. Intending to investigate a homogeneous patient cohort and to avoid deviations in measured parameters due to different biological tumor characteristics, we included *IDH* wild-type glioblastoma only, which resulted in a relatively small sample size, in particular, since our unique study design required both hybrid PET/MRI as well as an additional MRI for each patient.

Moreover, our study was unicentric and we only used two types of MRI scanners (Philips Achieva or Ingenia n = 36 and Siemens Verio n = 2). Due to the fact that the majority of our patients were scanned using a Philips MRI scanner, we received a homogeneous image data set. However, including various machines may lead to more reliable and generalizable results.

Our freely available in-house-developed BraTS Toolkit was utilized for automatic image processing and tumor segmentation [21]. Although the capability of this automatic data pipeline has already been investigated and all segmentations have been manually corrected when necessary, there is still the possibility of slight dissimilarities in these segmentations, which might have influenced our results. In addition, we needed to synthesize two missing T2w sequences to allow for automated segmentation, which might further influence segmentation. 

Furthermore, the majority of patients had a time interval between sequential PET and MRI examination of up to four weeks (92%). Consequently, our results account for this defined time span only. Exceeding this time interval comes with a higher probability of tumor growth or alterations in findings. Lastly, our findings need to be validated in an independent and greater patient cohort. 

## 5. Conclusions

We could not detect relevant differences in diagnostic performance between the simultaneous and sequential acquisition of FET-PET and DSC perfusion for patients with glioblastoma. Hence, sequential acquisition can be considered as clinically and scientifically acceptable within a time frame of four weeks, thereby reassuring routine clinical management. Our results provide crucial information about how advanced multimodal imaging can be implemented and validated in order to optimize personalized decision-making and improve outcomes in neuro-oncology.

## Data Availability

Patient data (MR and PET images) are not publicly available due to data privacy reasons.
